# Shallow Au implantation into silicon-on-insulator slot ring resonator waveguide devices

**DOI:** 10.1038/s41598-026-46478-x

**Published:** 2026-04-03

**Authors:** Quan-Shan Liu, Maddison Coke, Alexander Lincoln, William Wren, Tim Echtermeyer, Iain Crowe, Richard J. Curry

**Affiliations:** 1 Department of Electrical and Electronic Engineering, Photon Science Institute, University of Manchester, Oxford Road, Manchester, M13 9PL UK; 2 Electron Microscopy Centre, Faculty of Science and Engineering, University of Manchester, Oxford Road, Manchester, M13 9PL UK

**Keywords:** Materials science, Nanoscience and technology, Optics and photonics, Physics

## Abstract

The optical transmission spectra of a series of micro-ring resonators (MRRs) are studied following the implantation of gold (Au) ions and subsequent thermal annealing at temperatures between 500 $^\circ$C and 700 $^\circ$C. Whilst we find that this process leads to the ready formation of Au nanoparticles (NPs) partially embedded in the MRR surface, the cavity optical properties, Q-factor and extinction ratio (ER) are severely degraded, for annealing between 500 $^\circ$C and 600 $^\circ$C, but recover again for annealing at 650 $^\circ$C. For an equivalent (control) MRR, which received no Au implantation, thermal annealing alone was also found to degrade the cavity performance.

## Introduction

Silicon-based photonic devices have been a focus of intense research over recent decades due to their compatibility with the prevailing complementary metal-oxide-semiconductor (CMOS) fabrication technology^1–5^. Compared with other silicon photonics platforms such as silicon nitride^6^, silicon-on-sapphire^7^, suspended silicon^8^, and chalcogenides-on-silicon^9^, silicon-on-insulator (SOI) is the most mature and widely offered by a number of foundries^1^. As a result, researchers have developed a variety of SOI-based photonic building blocks^10,11^, among which microring resonators (MRRs) are well characterised and have been applied as functional sensors^12–20^, including via 2D materials integration^21–23^, and more recently for the realization of novel non-Hermitian photonics-based Parity-Time (P-T) symmetry structures^24,25^. Furthermore, the slot waveguide geometry has been introduced into MRRs for achieving enhanced light-matter interaction and thus improving sensing performance^26–29^.

The utilization of ion implantation techniques opens additional possibilities for the modification of material functionality^30^. This technique can be readily incorporated into CMOS processing with minimum impact on device fabrication^31^. Through the introduction of selected atomic species with well-defined doses and selected depths, implanted matrices may display novel properties. In particular, gold has been widely implanted into a range of targets including silicon wafers^32,33^, sapphire^34^, silicate glasses^35^, Nd: YAG crystals^36^, TiO$_{2}$ crystals^37^, LiTaO$_{3}$ crystals^38^, silica^39,40^, and polymethyl methacrylate resist^41^. It has been observed that with suitable post-implantation annealing Au nanoparticles (NPs) may be formed displaying plasmonic properties^36,42–45^. However, gold implantation directly into MRRs has remained unexplored, though some attention has been paid to other dopants including oxygen^46^, germanium^47,48^, silicon^49^, and boron^49,50^.

The formation of Au nanoparticles within or near the high-field regions of slot waveguides could combine localized surface plasmon resonances with the strong optical confinement of the slot geometry. Such hybridization may further enhance the local electromagnetic field intensity, providing improved sensitivity for various sensing applications^51–53^. In this paper we report experimental studies of gold implantation into SOI-based slot MRRs with the aim of Au NP formation and modification of the MRR optical response. Thermal annealing induces rapid Au diffusion in silicon, leading to Au NP formation predominantly near the waveguide surface, including some on the sidewalls. As a result, the Au NPs will be located close to the guided mode in the slot region leading to the possibility of some interaction occuring. The transmission spectra of MRR devices at each stage of processing (pre/post-implant and post-annealing) are measured to obtain extinction ratios (ER) and quality factors (QF) to assess the impact of the processing. Though in this study we found no evidence of any enhanced plasmonic effect associated with the coupling of Au NPs to the MRRs, the impact of the processing on the MRR optical response is elucidated.

## Sample details and methods

All the waveguide devices studied were rib-type and designed for single-mode operation around 1550 nm, with a 130 nm rib height and 90 nm slab thickness. Samples were fabricated using the CMC Microsystems Advanced Micro Foundry (AMF) silicon photonics process. The foundry fabrication process uses 193 nm deep UV lithography of a 220 nm SOI device layer on a 3 µm buried oxide (BOX) on a 600 µm handle wafer and includes both full and partial etch of the device layer, allowing for the incorporation of standard grating couplers for the delivery and collection of light, as well as standard TiN heater elements and associated electrical contact pads. The chip was coated with silica cladding of ~1.5 µm thickness for device protection.

### Device structure and ion implantation

**Fig. 1:**
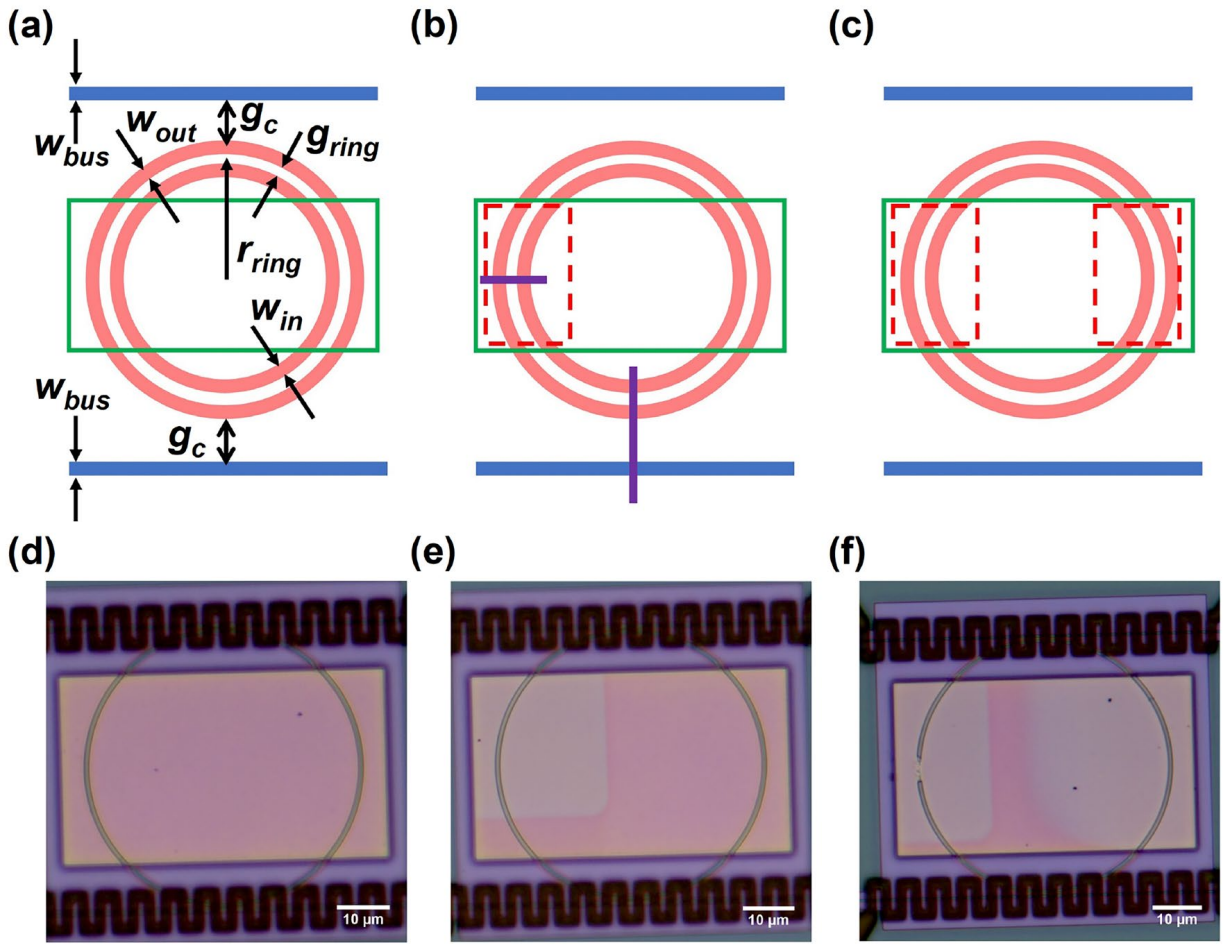
(**a**) to (**c**) Plan-view not to scale schematic illustration of three add-drop slot MRR devices with the device design parameters defined (values provided in the text). The blue lines represent the bus waveguides adjacent to the MRRs shown in pink. The etched cladding region is shown by the green box with the red dashed boxes indicating the regions implanted with Au ions. (**d**) to (**f)** Optical microscope images of each device post-implantation corresponding to (**a**) to (**c**). The implanted area is observed as the colourless region contrasting with the pink-tinged unimplanted regions. The black oscillating lines above and below the rings are integrated titanium nitride heater elements to enable potential thermo-optic tuning of the MRRs. These micro-heaters are positioned on the top oxide cladding over the bus-to-ring coupling regions.

The slot MRRs used in this study can be classified into two configurations consisting of (i) ‘all-pass’ and (ii) ‘add-drop’ designs. Figure 1a to c show schematic plan-views of three add-drop slot MRRs, with the structural parameters defined as follows: inner ring waveguide width $w_{in}$ = 290 nm; outer ring waveguide width $w_{out}$ = 250 nm; nearby bus waveguide width $w_{bus}$ = 320 nm; ring-to-ring gap $g_{ring}$ = 200 nm; ring-to-bus gap $g_{c}$ = 200 nm, 250 nm, and 300 nm for Figure 1a to c respectively; and radius to slot centre $r_{ring}$ = 25 µm. To allow ion implantation into the MRRs a series of 64 µm $\times$ 35 µm etched windows were opened through the silica capping layer using a selective hydrofluoric acid etch during the fabrication process, shown by the green box in Figure 1a-c and visible in the optical images in Figure 1d-f. The slot MRR device in Figure 1a remained unimplanted as a reference (referred to as device A); the two devices in Figure 1b and c were implanted with 25 keV Au+ ions into a region covering ~18$\%$ (Figure 1b, device B) and ~46$\%$ (Figure 1c, device C) of the total MRR with a dose of 5E14 ions/cm$^{2}$. In Figure 1b, two purple solid lines are positioned to indicate cross-sectional diagrams of the implanted region and bus-to-ring coupling region. (Supplementary Information, Figures S1a and S1b). Device C appears to have a discontinuity in its ring that was not noticed prior to characterization. Surprisingly, resonances are still measurable for this “incomplete-ring” structure, albeit with wider peak widths compared to those of devices A and B (vide infra).

A second set of single bus MRR devices were likewise implanted for study, again one of these all-pass slot MRR devices remained unimplanted providing a reference (referred to as device D). The two other devices (referred to as devices E and F respectively) were implanted with 25 keV Au+ ions into a region covering ~22$\%$ and ~49$\%$ of the total MRR respectively, with a higher dose of 1E15 ions/cm$^{2}$. The percentages of coverage are calculated based on the implantation areas presented in the optical figures, and the details of implantation of all the devices mentioned are summarized in Table 1.

**Table 1:** Summary of implantation details for each device.

Device	Implantation dose (ions/cm$^{2}$)	Comments
A	-	add-drop; reference device
B	5E14	add-drop; covering ~18$\%$ of the total MMR
C	5E14	add-drop; covering ~46$\%$ of the total MMR
D	-	all-pass; reference device
E	1E15	all-pass; covering ~22$\%$ of the total MMR
F	1E15	all-pass; covering ~49$\%$ of the total MMR

The ion implantation was performed using the platform for nanoscale advanced materials engineering (P-NAME) Facility (Ionoptika, Q-One) at the University of Manchester^41^. Inspection of the optical microscope images exhibits a clear contrast between implanted and unimplanted areas (clear and pink), illustrated in Figure 1d to f for devices A to C respectively. Post-implantation thermal annealing is typically required for both repairing ion damage and electronic activation of implanted dopants^54^. The selection of optimal annealing conditions is highly empirical and varies depending on the specific implanted species and the host material. In this case the temperature and duration must be sufficient to stimulate the migration of the implanted Au ions to form NPs whilst not resulting in damage to the waveguides. In order to determine a starting point for annealing whilst preserving MRRs, 25 kV Au+ was first implanted, at a dose of 5E15 ions/cm$^{2}$, into a simple on-chip linear waveguide along a 500 µm length. Annealing was then performed under a nitrogen atmosphere at 500 $^\circ$C. All the annealing mentioned in the text was completed using a rapid thermal annealer.

To ascertain that these annealing conditions are suitable for the formation of Au NPs SEM imaging of the implanted waveguides was undertaken (Supplementary Information, Figures S2a and S2b). Close inspection revealed the formation of Au NPs with a diameter of ~10 nm partially embedded in the sample surface. Energy dispersive x-ray spectroscopy (EDS) was used to compare waveguides with and without Au implantation with an enhancement of the Au x-ray signal observed (Supplementary Information, Figure S2c) in the implanted area. These results indicated that a 500 $^\circ$C anneal is a suitable temperature to initiate Au NP formation however, this alone may not be sufficient to repair implantation damage. To assess this the transmission spectra of the linear waveguide were measured pre- and post-implantation and again post-anneal. It is found that implantation leads to sufficient damage to prevent waveguiding which is not recovered by a 500 $^\circ$C anneal. Identical ion implantation and annealing procedures were also carried out on alternative SiN linear waveguides. However, SEM analysis revealed no observable formation of Au NPs on them, motivating the selection of the Si platform in this work.

To study the MRR devices A to F (Table 1) a series of thermal anneals were performed under a nitrogen atmosphere each lasting for 2 minutes at temperatures between 500 $^\circ$C to 700 $^\circ$C at 50 $^\circ$C increments, summarised in Table 2. The two unimplanted reference devices (A and D) were also subject to the same thermal annealing.

**Table 2:** Summary of annealing cycles undertaken.

Stage	Definition	Comments
1	Pre-implant	-
2	Post-implant	Devices A and D remained unimplanted
3	Post-anneal under 500 $^\circ$C	N$_2$ atmosphere for 2 minutes
4	Post-anneal under 550 $^\circ$C	N$_2$ atmosphere for 2 minutes
5	Post-anneal under 600 $^\circ$C	N$_2$ atmosphere for 2 minutes
6	Post-anneal under 650 $^\circ$C	N$_2$ atmosphere for 2 minutes
7	Post-anneal under 700 $^\circ$C	N$_2$ atmosphere for 2 minutes

**Fig. 2:**
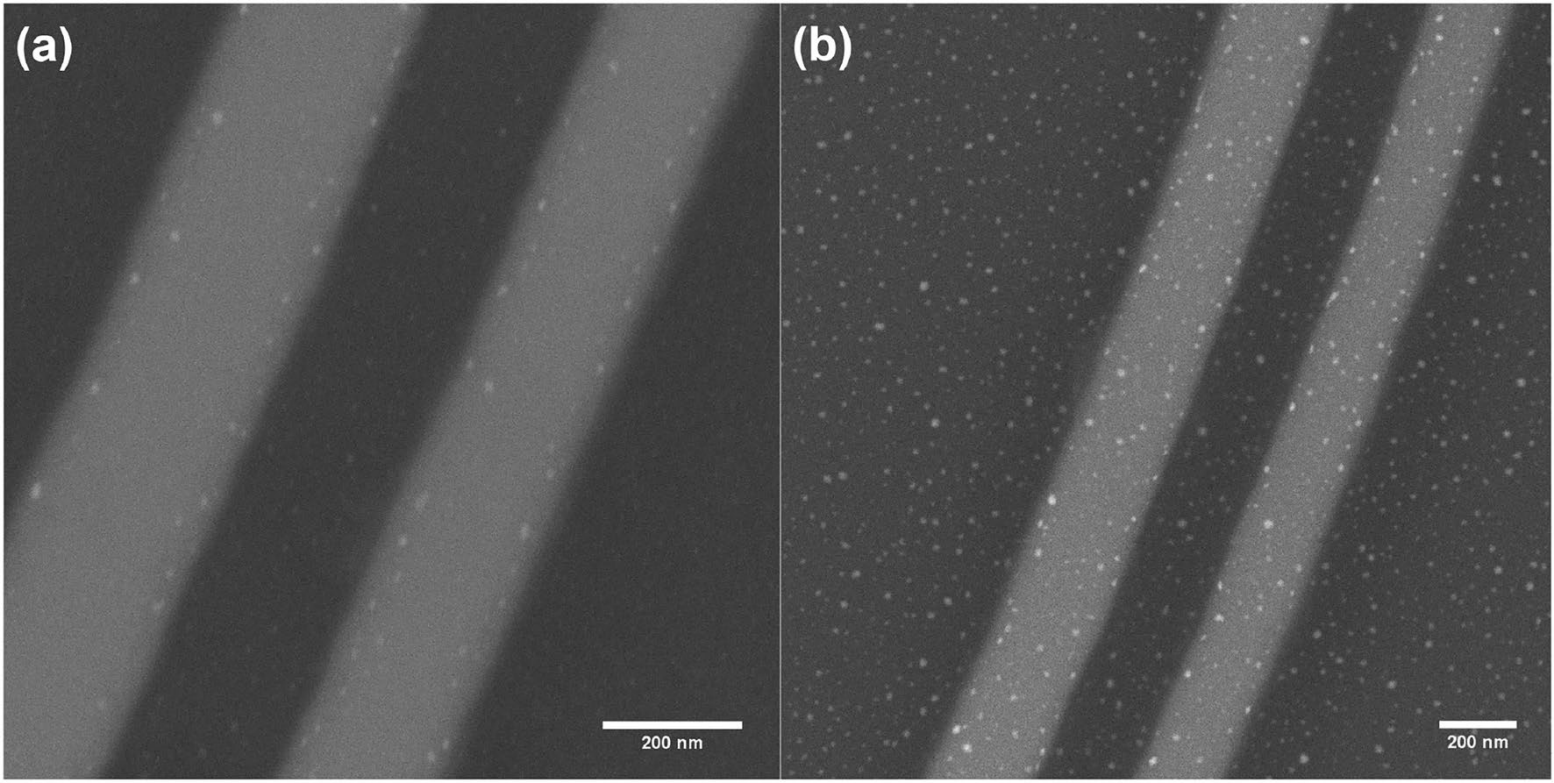
(**a**) Back scattered electron image for the implanted ring waveguide of device C. (**b**) back scattered electron image for the implanted ring waveguide of device F. Both devices have gone through a number of 2 min thermal anneals between 500 $^\circ$C and 700 $^\circ$C at 50 $^\circ$C intervals (stages 1 to 7 in Table 2).

Following each stage MRR characterisation was performed, described below, and post stage 7 SEM images of devices C and F were obtained (Figure 2). Within each image sporadic Au NP decoration can be observed, increasing in density with Au dose (5E14 and 1E15 ions/cm$^{2}$ for devices C and F respectively). Alongside this, EDS analysis was performed on the implanted and unimplanted areas of devices B and E (Supplementary Information, Figures S3a and S3b) showing an increase of the Au signal in the implanted regions.

## Experimental set-up

To quantify the impact of the Au implantation and thermal annealing on the MMR devices the transmission of each device’s bus waveguide was measured at each stage of processing. For the add-drop slot MRRs, the same bus waveguide was consistently selected for characterization after each stage. A Thorlabs 1550 nm fibre-coupled benchtop superluminescent diode coupled to a single-mode cleaved-end fibre was used to launch light into the waveguide via the predefined grating couplers. Transmitted light was similarly collected using a multi-mode fibre and inserted into an Anritsu optical spectrum analyser (OSA). The light source’s broad emission spectrum enables rapid acquisition of the full transmission response across multiple resonances without the need for wavelength scanning, (e.g., with a tuneable laser) which is particularly useful for observing overall spectral changes. While the limited coherence can reduce spectral contrast, it does not affect the accurate identification of resonance patterns or changing trends in ER and QF. All measurements were taken at room temperature and over the 1450 nm to 1650 nm spectral range.

The measured input and output wavelength dependent power ($P_{in}$ and $P_{out}$ respectively) is used to obtain the transmission loss of waveguides (including input and output grating couplers), as:

1 $$\begin{aligned} Loss = 10\times log_{10}(P_{out}/P_{in}) \end{aligned}$$

**Fig. 3:**
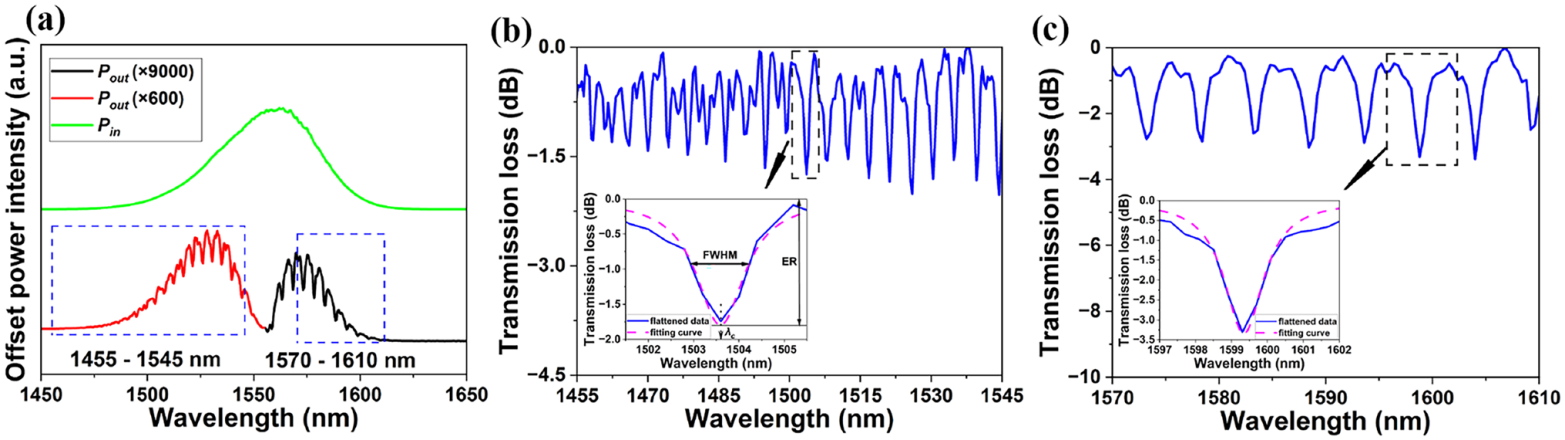
(**a**) The input and output power intensity of reference device A as received (vertically offset for clarity). Different parts of the output spectrum have been respectively enlarged by a factor of 600 (red curve) and 9000 (black curve) for clarity. The two selected regions over which background subtraction and analysis was performed are shown enclosed by blue dashed boxes. (**b**) and (**c**) Demonstration of spectrum flattening and resonance peak fitting for the two selected regions displayed in (a).

Figure 3a shows the $P_{in}$ and $P_{out}$ of the unimplanted reference device A during one measurement. Each spectrum was collected three times, performing a fresh fibre alignment each time, to obtain the mean value and standard deviation.

## Data processing and analysis

In Figure 3a, the grating coupler bandwidth is not in perfect alignment with the light source. Nevertheless, resonant patterns are observed both below and above 1550 nm. As such, two selected regions (1455 to 1545 nm, and 1570 to 1610 nm) of each measured spectra were chosen for data processing and analysis, each displaying multiple resonance peaks. The processing removed the background response within the selected regions using subtraction of a polynomial fit resulting in a flattening of the spectrum, Figure 3b and c. This step does not change any resonance properties and is helpful for resonance comparison among different spectra. The ER and QF were derived from Lorentzian fitting of the resonance peaks based on the least square method using MATLAB codes modified from that provided in reference^55^.

Figure 3b and c show the flattened data (blue line) and the fitted Lorentzian curves to one resonance (magenta dashed line) for the two marked regions in Figure 3a. Relevant parameters including one peak’s ER, full-width half maximum (FWHM), and central resonant wavelength ($$\lambda$$$$_c$$) are illustrated in Figure 3b inset. Separate spectrum flattening and curve fitting is completed for the two selected wavelength regions. To ensure consistent convergence of peak fitting, only resonances exceeding 70% of the deepest resonance peak in each region are selected for fitting.

## Results and discussion

### Transmission spectrum analysis

**Fig. 4:**
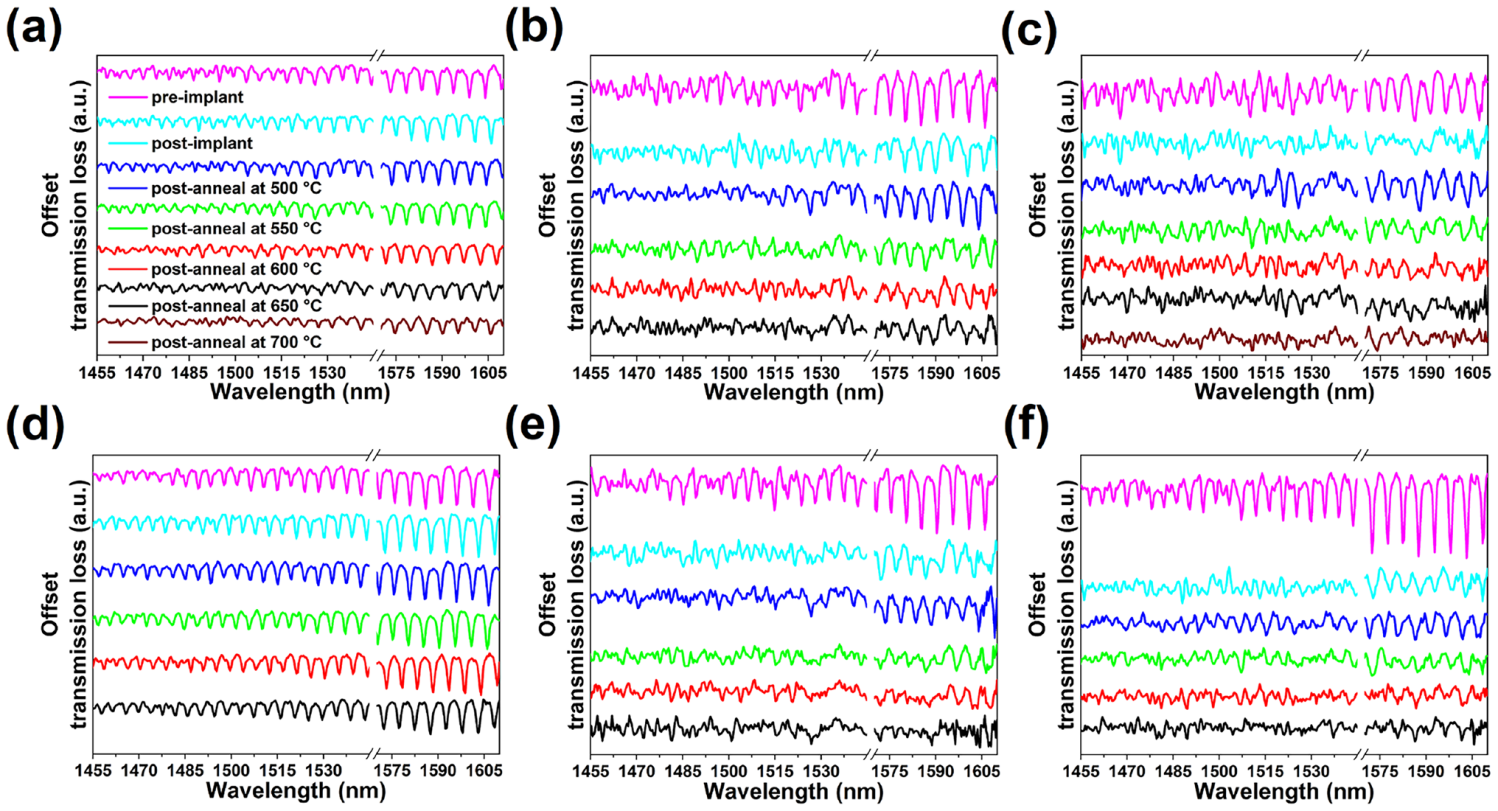
(**a**) to (**f**) Flattened transmission spectra of devices A to F respectively. Within each panel the stages progress from top (stage 1) to bottom as indicated in (**a**) and are vertically offset for clarity.

Figure 4a to f show the flattened resonance peaks of devices A to F (Table 1) respectively at all stages of processing (see Table 2 for stage definition). The transmission of devices B, D, E, and F following stage 7 (700 $^\circ$C annealing) are not included as these devices exhibited no transmission. This clearly indicates device failure resulting from thermally induced stress relief in the top SiO$_2$ cladding that results from hydrogen released during decomposition of SiOH bonds in the SiO$_2$^56,57^. Damage can be visibly observed by comparing optical microscope images of the devices following stage 1, 6, and 7 (Supplementary Information, Figure S4).

Following implantation (stage 2) but prior to any annealing, all implanted devices experience a reduced coupling to the MRR with devices C and F (higher doses) reducing more than devices B and E (lower doses). The QFs of both of the unimplanted reference devices are also found to be slightly different which might be attributed to variation in laboratory conditions (e.g. humidity) between measurements. Following 500 $^\circ$C annealing it is observed that the MRR resonance peaks have recovered somewhat, indicating a reduction in ion-induced damage. Additionally, at this temperature Au ions have diffused out of the waveguides to form Au NPs partially embedded in the device surface as shown in Fig. 2. Annealing at higher temperature (stages 3 to 7) results in a general degradation of the MRR ER. For the reference unimplanted devices, a slight degradation in MRR QF can be seen as annealing progresses. To quantify the above outlined impact of annealing on the devices Lorentz fitting of the resonance peaks was performed.

### Extinction ratio analysis

**Fig. 5:**
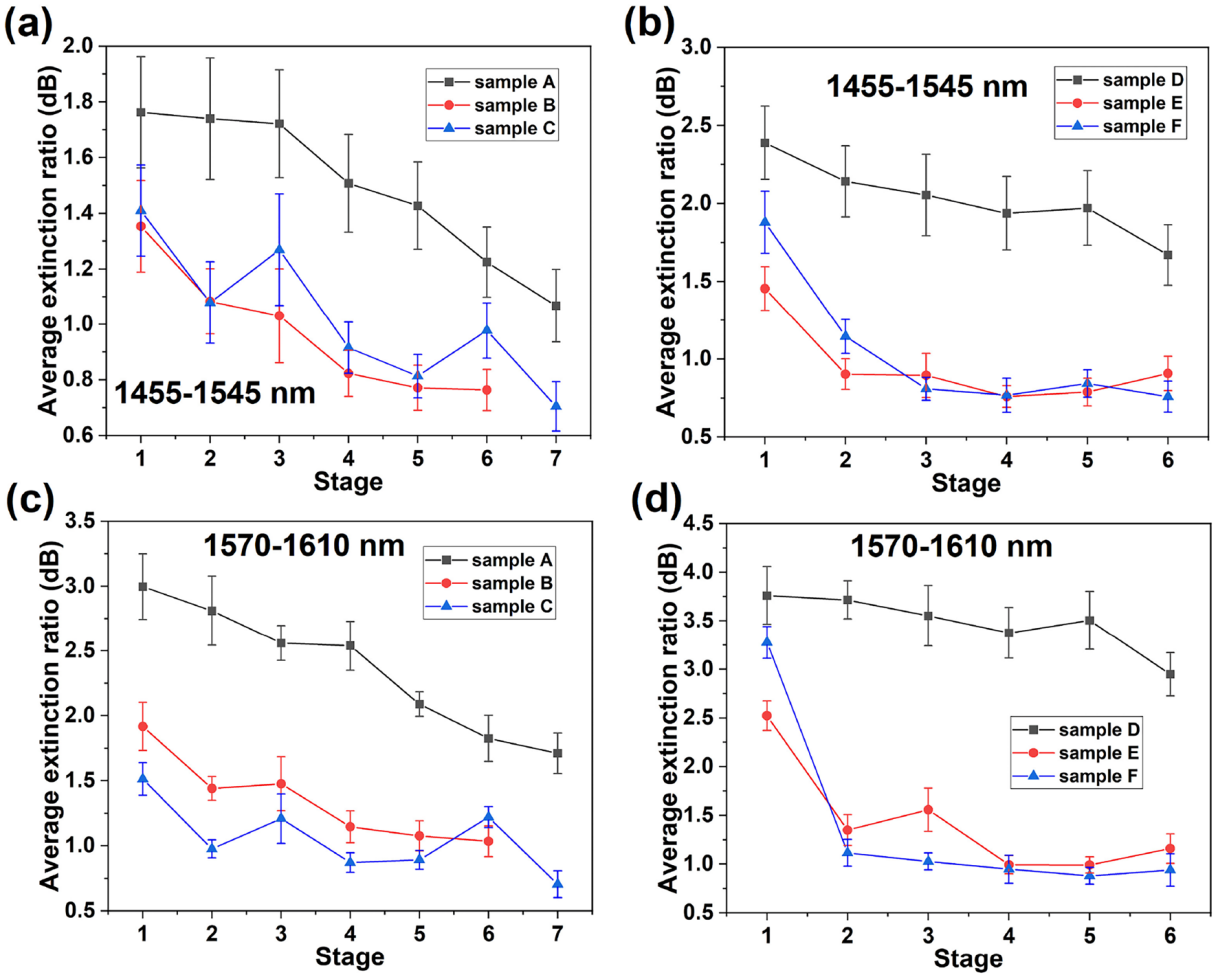
The average ERs of (**a**) devices A to C in the 1455 to 1545 nm region, (**b**) devices D to F in the 1455 to 1545 nm region, (**c**) devices A to C in the 1570 to 1610 nm region and (**d**) devices D to F in the 1570 to 1610 nm region following each experimental stage (Table 2).

To extract ERs from the resonance peaks displayed in Fig. 4, Lorentz curve fitting was performed as described above. Figure 5 displays the average ER within each wavelength region analysed for each of the different experimental stages performed, with error bars representing the standard deviation.

As shown in Figure 5a and b, the average ERs measured across the 1455 nm to 1545 nm region of devices B, C, E, and F reduce by 20$\%$, 24$\%$, 38$\%$, and 39$\%$ following implantation. A similar trend is displayed within the 1570 nm to 1610 nm region, Figure 5c and d, but with greater reduction of the ERs by 25$\%$, 36$\%$, 47$\%$, and 66$\%$ for devices B, C, E, and F respectively. The reduction in ER increases with implantation dose and area, strongly linking it to ion implantation induced damage within the MRRs. Annealing at 500 $^\circ$C does not repair this damage as mentioned above for the test linear waveguide, however Au NPs are partially embedded in the MRR surface. Measurement of the bus waveguide transmission spectra before and after implantation, and following 500 $^\circ$C annealing, did not reveal any obvious spectral difference. Plasmonic coupling to the surface plasmon modes associated with Au NPs is ruled out as the wavelengths employed here (around 1550 nm) are far from the expected Au NP surface plasmon resonances (515 nm to 570 nm for 10 nm to 100 nm diameter NPs respectively)^58^.

To see if higher annealing temperatures can be effective in recovering performance the further sequential anneals were performed up to 700 $^\circ$C. Following each of the subsequent annealing stages the ERs of both of the unimplanted reference devices exhibit a steady reduction. Those of the implanted devices B and C likewise continue to decline with post implantation annealing. In contrast the ERs of devices E and F appear to remain similar to their post implantation values. High-temperature annealing is expected to lead to some structural deformation due to the stress relief within the chip resulting in increased loss. However, annealing also recovers ion-induced damage and can lead to surface smoothing which should reduce loss. These effects are in competition and would appear to be balanced for devices E and F.

### Quality factor analysis

**Fig. 6:**
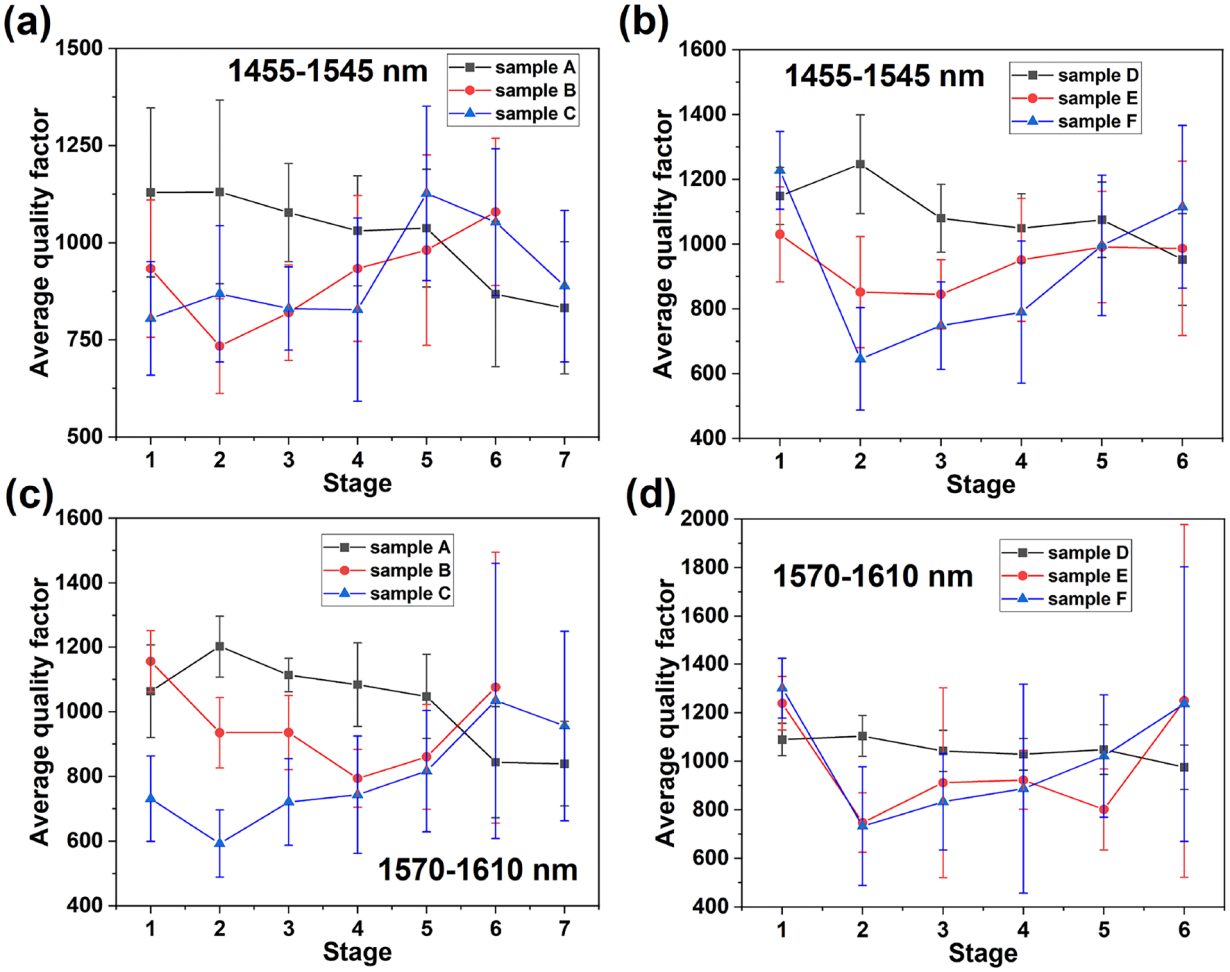
The average QFs of (**a**) devices A to C in the 1455 to 1545 nm region, (**b)** devices D to F in the 1455 to 1545 nm region, (**c**) devices A to C in the 1570 to 1610 nm region and (**d**) devices D to F in the 1570 to 1610 nm region following each experimental stage (Table 2).

The quality factor (QF) is defined as the ratio of the central resonant wavelength to the full-width half maximum of the resonance. High QFs are usually desired to realise ultra-sensitive photonic devices. QFs were extracted from the Lorenzian fits to the resonance peaks shown in Fig. 4. Figure 6 presents the average QFs of each device at each experimental stage, with the error bars indicating the standard deviations. The OSA resolution was 0.947 nm, limiting the precise determination of absolute QFs above 1000. Nevertheless, since all measurements were performed under identical conditions, the relative trends and variations remain reliable.

The variation of the QFs with implantation and annealing is not as obvious as that of the ERs with most variation occurring within the measurement error confidence. For the unimplanted devices, Figure 6a and b suggest that the QF of reference device A undergoes only a small reduction until an obvious decrease at stage 6 (annealing at 650 $^\circ$C). The QF of reference device D is barely affected by annealing. Following ion implantation, a clear reduction in QF is generally observed for all devices as shown in Fig. 6. There appears to be no strong relationship between the magnitude of the QF reduction and implantation dose or area. As annealing proceeds up to 650 $^\circ$C, the overall outcome is for the QFs of implanted devices to return to a similar value as that measured pre-implantation. Further annealing at a higher temperature (700 $^\circ$C, stage 7) does not seem to result in any further QF increase but did damage several devices. Further analysis is impeded by relatively large error bars which are possibly caused by the uneven distribution of peak widths indicated in Fig. 4.

## Conclusion

We have explored the feasibility of forming Au NPs partially embedded in the surface of SOI slot MRR waveguides through the use of FIB implantation. The presence of the Au NPs following 25 keV Au implantation at doses of 5E14 and 1E15 ions/cm$^2$ and following a 500 $^\circ$C thermal anneal was verified via SEM imaging and EDS analysis. No evidence of plasmonic coupling between slot waveguides and Au NPs was found, as the operating wavelengths used to measure transmission (centred at 1550 nm) are far from the expected resonance of the Au surface plasmons (515–570 nm for 10–100 nm particles). The impact of ion implantation on the waveguiding properties of the MRR systems is to reduce their cavity ER and QF, as would be expected due to ion induced damage. Thermal annealing alone, at temperatures from 500 $^\circ$C to 700 $^\circ$C, is also observed to result in a reduction of the waveguiding and MRR performance indicating that damage is occurring which we attribute to stress relief within the structures. Annealing of the Au implanted devices is found to result in a further reduction in waveguiding as measured by the ERs of the measured devices. In contrast annealing at a temperature of 650 $^\circ$C was able to almost fully recover the measured QFs of implanted devices. Whilst this work demonstrated the ability to form Au NPs through direct writing of Au ions into MMR devices, the resulting optical properties are degraded by both the implantation itself and the thermal treatment.

## Supplementary Information

Supplementary Information.

## Data Availability

The datasets used and/or analysed during the current study are available from the corresponding author upon reasonable request.
