# Supplementary material for: Shallow Au implantation into silicon-on-insulator slot ring resonator waveguide devices
Source: Sci Rep. 2026 Apr 3;16:15959. doi: 10.1038/s41598-026-46478-x (PMC13195044; doi:10.1038/s41598-026-46478-x)
Supplement: Supplementary file 1 — Supplementary Information. [file 41598_2026_46478_MOESM1_ESM.pdf]

Shallow Au implantation into silicon-on-insulator slot ring resonator waveguide devices: supplementary information

QUAN-SHAN LIU[1], MADDISON COKE[1], ALEXANDER LINCOLN[2], WILLIAM WREN[1], TIM ECHTERMEYER[1], IAIN CROWE[1], AND RICHARD J. CURRY[1,*]

[1] *Department of Electrical and Electronic Engineering, Photon Science Institute, University of Manchester, Oxford Road, Manchester, M13 9PL, UK*
[2] *Electron Microscopy Centre, Faculty of Science and Engineering, University of Manchester, Oxford Road, Manchester, M13 9PL, UK*
[*] *richard.curry@manchester.ac.uk*

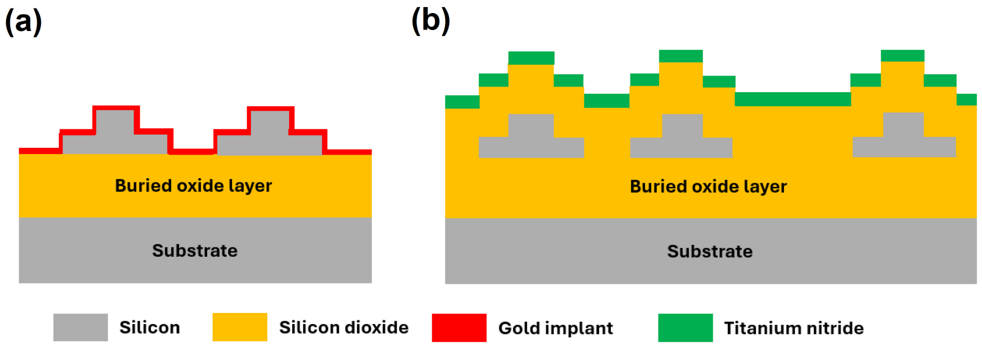

Fig. S1. Cross-sectional diagrams of the (a) implanted region and (b) bus-to-ring coupling region.

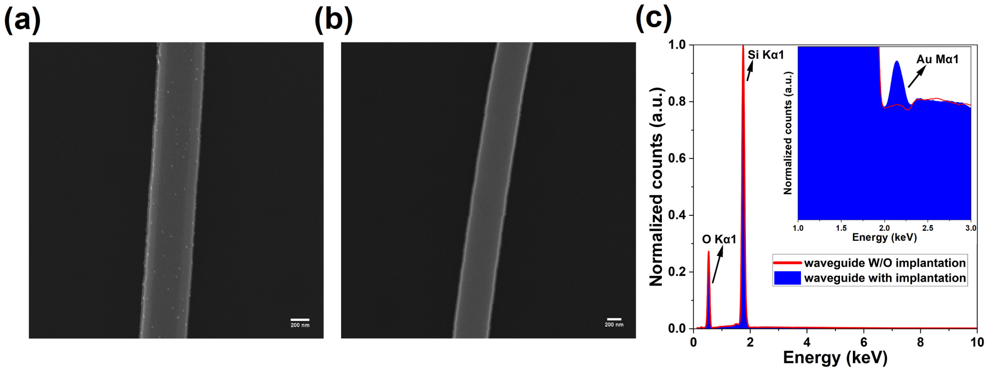

Fig. S2. Post-annealing (500 °C) secondary electron images of waveguides (a) following 5E15 cm^{-2} gold implantation, (b) without gold implantation. (c) EDS spectra of the devices mentioned in (a) and (b) showing clear increase in Au signal in the implanted device.

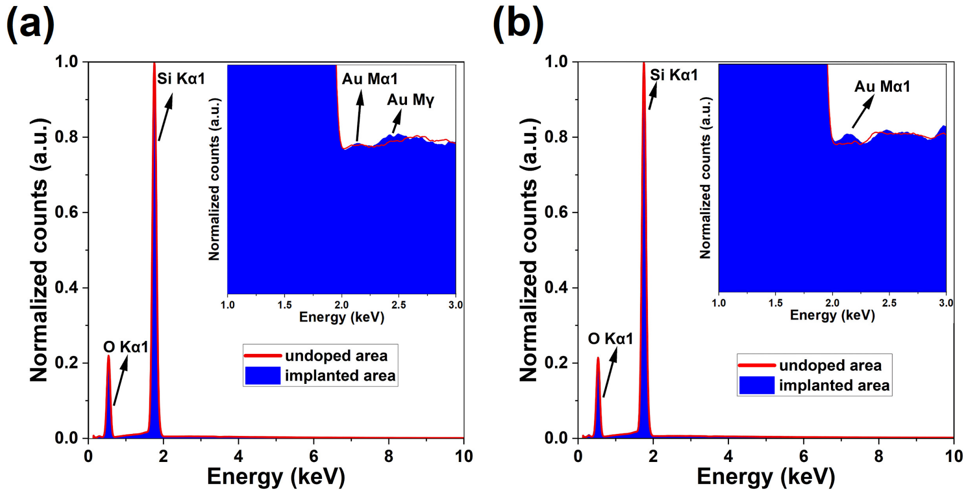

Fig. S3. Post-annealing EDS spectra for the unimplanted and Au implanted areas of (a) device B, (b) device E.

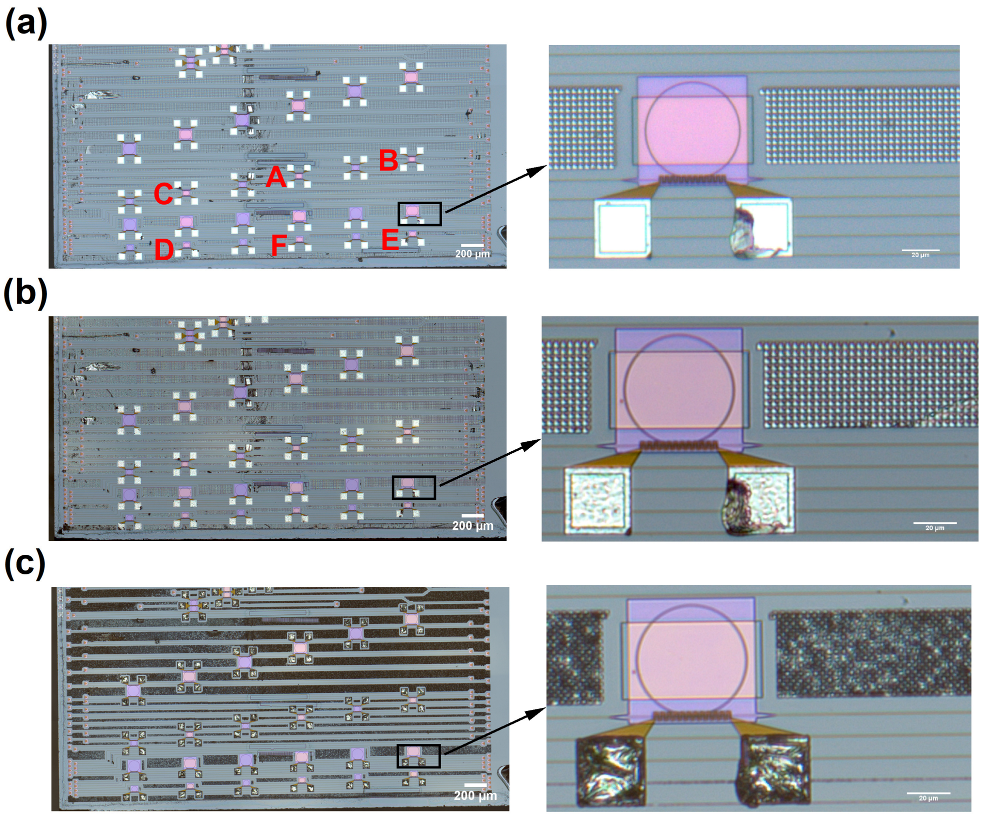

Fig. S4. Optical images of the photonic chip showing devices studied, labeled to the left of each MRR. (a) Image taken prior to implantation. (b) Image taken following implantation (except reference devices A and D) and a series thermal anneals from 500 °C to 650 °C. (c) Image taken following a further anneal at 700 °C. In (c) it can be observed that the chip has been thermally damaged by the contrasting dark regions with respect to (a) and (b). Those pixilated regions shown in zoom-in views are filled with aluminium dummy blocks and are not connected to any waveguide devices. It is hypothesized that the colour contrast was caused by the refractive index change due to surface oxidation.
